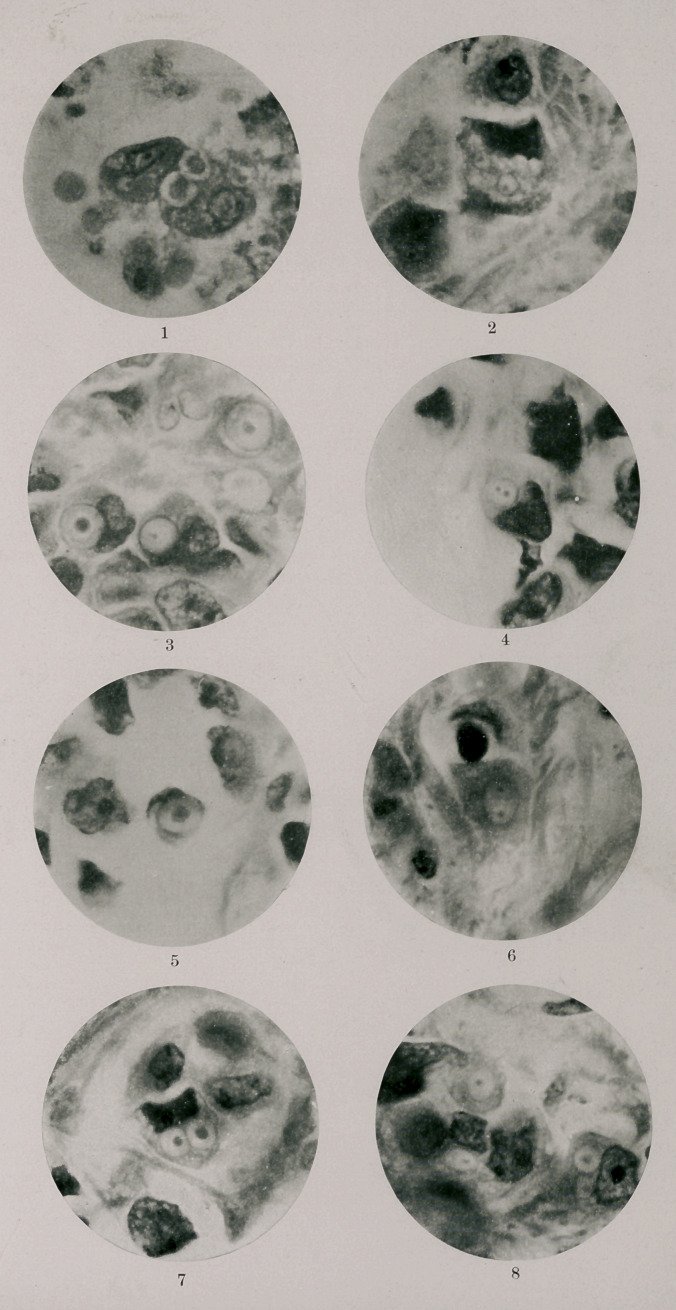# On the Evidence of Nuclear Division of Certain Cell Inclusions (Thoma, Sjöbring, Plimmer’s Bodies) in Cancerous Epithelium

**Published:** 1903-05

**Authors:** Harvey R. Gaylord

**Affiliations:** Director


### SPECIAL ARTICLE.

### On the Evidence of Nuclear Division of Certain Cell Inclusions (Thoma, Sjöbring, Plimmer's Bodies) in Cancerous Epithelium.

**From the Cancer Laboratory of the New York State Department of Health, University of Buffalo.**

By HARVEY R. GAYLORD, M. D., Director.

CONTENTS.

Introduction.
History of the bird's-eye inclusions in cancer.
Theories advanced as to the significance of the bodies.
(*a*) As degenerations.
(*b*) As parasites.
Comparison of the bird's-eye inclusions with the ameboid form of *Plasmodiophora Brassicæ*.
Appearance and methods of division of *Plasmodiophora Brassicæ* in the plant cell.
Evidence of a process of division in bird's-eye inclusions.
Similarity of spores of *Plasmodiophora* with certain forms of bird's-eye inclusions.
Evidence from the literature of nuclear division of inclusions.
Conclusions.
Description of plates.

SINCE the advent of cellular pathology the attention of investigators has been directed to certain bodies embedded in the cytoplasm of the cells of malignant tumors. The difficulty of determining the nature of these inclusions is well evidenced by the extensive literature pertaining to the subject, but which even today leaves the significance of at least a number of these structures in doubt.

The bodies with which we wish to deal in this paper are spherical or slightly oval structures from .004 to .01 mm. in size, or in exceptional cases of still greater dimensions. They invariably contain a small body usually centrally placed. With the exception of this central body the resting inclusions present an appearance not unlike that of a vacuole, as no structure other than the central body is demonstrable. The entire structure is embedded in the protoplasm of the cancer cells and, where the body is of greater dimension, the cell nucleus is commonly pushed to one side and curved about it. The staining reaction of these inclusions is dependent largely upon the fixative employed. In material hardened with formalin and stained with Delafield's or iron hematoxylin, the central bodies of a certain number of the inclusions take the nuclear stain. Where osmic acid is used as a fixative, in which case the inclusions are most definitely demonstrated, the central body of the inclusions stains as does the protoplasm. These bodies may be found unstained in fresh material directly after removal of the tumor.

Although, as will be seen from the historical review, these bodies have been repeatedly observed and in many cases accurately illustrated, they have in recent times through the exhaustive article of Plimmer attracted renewed interest. In several of the recent publications on the subject, the bodies have been spoken of as "Plimmer's bodies." Von Leyden, one of the most recent writers on the subject, has called attention to their similarity in appearance to the eye of a bird, for which reason they are coming more and more to be spoken of as "bird's-eye inclusions," a not inapt expression.

*Historical.*—On review of the literature it becomes evident that many observers have seen and illustrated the inclusions above described. As early as 1847, in the first volume of Virchow's Archiv. is found an article by Virchow, entitled, "Zur Entwickelungsgeschichte des Krebses, nebst Bemerkungen über Fettbildung im thierischen Körper und pathologische Resorption," in which on Plate II., Fig. 5, the cells shown at *a*, *g*, *i*, *l*, *n*, and *k*, undoubtedly contain inclusions of this nature. It is interesting to note that the author represents cells in the fresh state. Virchow interpreted these bodies as modifications of the nuclei of the cancer cells.[1]

---

1. The cell inclusions described by Langhans in *Deutsche Chirurgie von Billroth und Lücke*, 1886, in the epithelial cells of carcinoma of the glands of Kupfer, do not belong under this heading, although Lubarsch (*Pathologische Anatomie und Krebsforschung*) wishes so to classify them. The bodies described and illustrated by Langhans are round, well-defined bodies, from the size of a nucleus to that of a large vacuole. They were, however, filled with granular material and surrounded by deeply stained membranes. Many of them were drawn out in tubular or branching form. It will be seen that this description in no way conforms to that of the bird's-eye inclusion.

In Vol. III. of Virchow's Archiv. (Die endogene Zellenbildung beim Krebs) Virchow further describes cell inclusions of which those illustrated on Plate II., Figs. *2c*, *4a*, 2 and *d* appear to represent bird's-eye inclusions, although the illustrations are not sufficiently characteristic to remove a possible doubt. (See Fig. 1.)

FIG. 1.

Plate II., Fig. *4d*, after Virchow.

Soudakewitsch[1] in two articles published in 1892, gives repeated illustrations of these bodies. Plate V., Fig. 6, carcinoma of breast, represents a body apparently undergoing division with four central bodies arranged in the form of a cross. Fig. 2, Plate VI., also carcinoma of the breast, shows multiple inclusions, the one above and to the right presenting a definite phase of division. (See Fig. 2.) Fig. 27, Plate VI., carcinoma of the breast, hardened in Fleming and stained with safranin, represents a resting body. Plate VII., Fig. 17, carcinoma of the pancreas, hardened with osmic acid and stained with hematoxylin, represents a rosette form. Fig. 19 represents a cell with multiple inclusions from the metastases of cancer of the pancreas. In a second article in August of the same year he describes further inclusions. That of Fig. 7, Plate XII., from a primary case of carcinoma of the pancreas, illustrates seven definite inclusions, with central bodies forming a rosette. (See Fig. 2.) Fig. 15, from a primary carcinoma of the liver, stained with osmic acid and hematoxylin, represents a group of these bodies, and in Fig. 17,

FIG. 2.

*a* Plate XII., Fig. 17, after Soudakewitsch.

*b* " VI., " 2, " "

*c* " XII., " 7, " "

1. Soudakewitsch.—Recherches sur le parasitisme intracellulaire, etc. *Annales de l' Institut Pasteur*, 1892, March 25, No. 3.

a body undergoing division is represented from a carcinoma of the pancreas. (See Fig. 2.) Fig. 21 again represents a group of similar inclusions from a case of cancer of the liver.

FIG. 3.

*a* Plate IV., Fig. 1, after Sjöbring.

*b* " IV., " 14, " "

Sjöbring[1] gives a most accurate and consistent description of bird's-eye forms of inclusions. In Figs. 1, 12 and 14 he illustrates characteristic forms. (See Fig. 3.) Thoma[2] describes bodies somewhat similar to those of Sjöbring. His original article is unaccompanied by illustrations. Illustrations given in his Lehrbuch f. path. Anatomie, 1894, p. 169, leaves some doubt as to the identity. Fig. *a* apparently represents a bird's-eye inclusion, and those shown in the large figure above likewise appear to be of this nature. Figs. *c* and *d* of this illustration, representing a form of division, do not coincide with any forms we have personally observed. (See Fig. 4.)

FIG. 4.

*a* Adenocarcinoma of the rectum—Thoma.

*b* Parasitic forms from above.

1 Sjöbring.—Ein parasitäre Protozoartiger Organismus in Carcinomen. *Fortschritte d. Medecin*, 1890, p. 529.

2. Thoma.—Ueber eigenartige parasitäre Organismen in den Epithelzellen der Carcinome. *Fortschritte der Medecin*, 188), p. 413.

An investigator who undoubtedly observed these bodies was P. Foa.[1] (See Fig. 5.) In Figs. 2 and 16 of Plate II. and 13 of Plate III., this author illustrates these bodies from carcinoma of the breast. He also recognises the identity of many of the bodies described by him and those of Soudakewitsch, just mentioned.

Kürsteiner[2] describes two cases of carcinoma, one villous carcinoma of the bladder and the other papillary adenocarcinoma of the uterine mucosa, in which he found embedded in the cells spherical and oval bodies with well-defined central structures staining with eosin. His illustrations are all taken from a villous carcinoma of the bladder and leave no doubt that in Figs. 3, 4, 5, 6, 7 and 8, Plate XII. he was dealing with typical bird's-eye inclusions. (See Fig. 6.) Of interest are the large number of inclusions which Kürsteiner noted in certain cells of his preparations. In one cell he was able to count as many as *eighty inclusions*. He inclines to the belief that the inclusions may be parasites, but expresses himself with great caution.

FIG. 5.

*a* Plate II., Fig. 2, after Foa.

*b* " II., " 16, " "

Seegenbeck van Heukelom,[3] in an address before the 10th National Medical Congress in Berlin, 1890, describes the presence in carcinoma, including nearly every type, covering some 200 different cases, of large and small spherical inclusions in carcinoma cells. These sometimes presented a double contour, appeared to be filled with protoplasm and presented a nuclear-like structure, which stained with carmine. Besides these so-called larger spheres he describes smaller ones, which he says were found embedded in the protoplasm and in the nuclei. They did not stain with carmine, and but weakly with eosin. They were not very refractive. From his description of the larger bodies, it is certain that they corresponded with those of Wickham, and of

1. Foa.—Ueber den Krebsparasiten. *Cent. f. Bakteriologie*, Vol. XII., p. 185, 1892.

2. Kürsteiner.—Beiträge zur pathol. Anatomie der Papillome und papillomatösen Krebse von Harnblase und Uterus. *Virchow's Archiv.*, Vol. CXXX., p. 463.

3. Seegenbeck van Heukelom.—*Centralblatt f. allg. Pathologie und pathologische Anatomie*, Vol. I., p. 204.

the smaller it is difficult to say whether they represent the typi-

Fig. 6.

*a*

*a* Plate XII., Fig. 3, after Kürsteiner.

*b* " XII., " 6, " "

cal bird's-eye inclusions or not. The description is, unfortunately unaccompanied by illustrations.

Fig. 7.

Plate VII., Fig. 2, after Podwyssozki and Sawtschenko.

The description given by Podwyssozki and Sawtschenko[1] varies somewhat from the description of other authors but some of the inclusions illustrated by them present the characteristics of the typical bird's-eye form. Fig. 2, Plate VII.; Fig. 20, Plate VIII., inclusion to the left, are characteristic. (See Fig. 7.) The technique employed by these authors was somewhat different from that of others, although based on osmic acid fixation, the stain being safranin and picric acid.

1. Podwyssozki and Sawtschenko.—*Centralblatt f. Bakteriologie*, Vol. XI., No. 16, 1892, p. 493.

After a most careful review of the forms of inclusions previously described in carcinoma, Borrel[1] concludes that the only bodies which under any circumstances could be considered as parasitic are the typical bird's-eye inclusions. In Figs. 8 and 9, Plate II., Borrel illustrates two large epithelial cells from epithelioma of the maxillary region, containing typical bird's-eye inclusions. (See Fig. 8.) Fig. 9 of this plate likewise shows forms suggestive of nuclear division, to be considered later.

FIG. 8.

Plate II., Fig. 9, after Borrel.

Sawtschenko[2] describes as appearing in several tumors, the origin and number not being given, cell inclusions of the bird's-eye type. Those shown in Figs. 7, 8, 9, 10 and 11 are entirely characteristic. (See Fig. 9.) His preparations were from material hardened in Flemming's solution, stained with safranin.

Ruffer and Walker[3] illustrate these inclusions in Plate XIV., Figs. 4 and 8, from two cases of gastric carcinoma; Plate XVI., Fig. 10, from carcinoma of the breast; Figs. 20, 21, 25, 28, 29, hardened with Flemming and stained with methyl-green, Biondi, also from cancer of the breast.

FIG. 9.

*a* Plate I., Fig. 10, after Sawtschenko.

*b* " I., " 9, " "

Ruffer and Plimmer[4] in Figs. 2, 5, 6, 12, 18, 32, 35, 37 and 49 of Plates I., II., and III., give excellent illustrations of typical inclusions. (See Fig. 10.)

1. Borrel.—Evolution cellulaire et parasitisme dans l'epithelioma. *Montpellier*, 1892, 40. *These*.

2. Sawtschenko.—Ueber schmarotzende Sporozoen in Krebsgeschwülste. *Centralblatt f. Bakteriologie*, Vol. XII., 1892, p. 17.

3. Ruffer and Walker.—On some parasitic protozoa found in cancerous tumors. *Journal of Pathology and Bacteriology*, 1893, p. 198.

4. Ruffer and Plimmer.—Further researches on the parasitic protozoa found in cancerous tumors. *Journal of Pathology and Bacteriology*, 1894, p. 3.

J. Jackson Clarke[1], illustrates on Plate III., Fig. 6, a group of typical bird's-eye inclusions, and at 7 and 10, masses of what

FIG. 10.

*a* Plate I., Fig. 5, after Ruffner and Plimmer.

*b* Plate XXIII., Fig. 11, after Ruffner and Plimmer.

appear to be spore-like bodies of a similar nature. (See Fig. 11.) He considers these inclusions to be sporozoa.

C. H. Cattle[2] describes typical bird's-eye inclusions and

FIG. 11.

*a* Plate III., Fig. 10, after J. Jackson Clarke.

*b* " III., " 7, " " "

*c* " III., " 6, " " "

especially calls attention to the fact that the inclusions can be found in the acini of the mammary gland at the margin of cancer of the breast, at the points where the epithelium of the acini

1. J. Jackson Clarke.—Observations on the Histology of cancer. *Centralblatt f. Bakteriologie*, Vol. XVI., 1894, p. 281.

2. Cattle, C. H.—Observations on the Histology of Carcinoma and the Parasite-like Bodies found in them. *Journal of Pathology and Bacteriology*, Vol. II., p. 367, 1894.

is undergoing cancerous transformation. This is probably the first definite reference to the presence of bird's-eye inclusions in the epithelium of the acini, where the epithelium still maintains its typical acinous arrangement. (See Fig. 12.)

Fig. 12.

Plate XXII., Fig. 9, after Cattle.

Sawtschenko[1] again describes, under a number of cell inclusions of varying forms, certain that represent typical bird's-eye inclusions. Fig. 30, Plate VI. (see Fig. 13), carcinoma of the breast and 32 from the same; Plate V., Fig. 24, the lower figure, from carcinoma ventriculi, and 25, upper figure, carcinoma of the breast, are clearly defined bird's-eye inclusions.

Pianese[2] shows in Fig. 2, Plate IV. (see Fig. 14), in one of the cells to the left, well-defined bird's-eye inclusions from carcinoma of the breast.

Fig. 13.

Plate VI., Fig. 30, after Sawtschenko.

F. J. Bosc[3] shows in Plate I., Figs. 1, 2, 3, 4, 5, 6, 7, 10, 11 and 38*b*, well-defined bird's-eye inclusions. On Plate II., Fig. 1 (see Fig. 15), are many inclusions of this type. Fig. 2*m*, Fig. 3*x*, Fig. 4*m*, Fig. 1*a*, Fig. 2*m*, Fig. 5*m*, 14, 15, 17 and 18 are from a case of carcinoma of the pancreas and liver. Plate IV., Figs. 26 and 28, epithelioma of the lower lip. On Plate II., Fig. 4 at "mor"; Plate III, Figs. 14, 15, 16, 18 and 19; Plate IV., Fig. 28; Plate V., Fig. 8 at "mor"; Fig. 12 at "mor"; Plate VII., Fig. 2*b* and Plate X., Fig. 4, the author pictures closely arranged groups

1. Sawtschenko.—Sporozoen in Geschwülste. *Bibliotheka Medica*, DII., Part 4, 1895.

2. Pianese. Beitrag zur Histologie und Aetiologie des Carcinoms. *Ziegler's Beiträge*, 1896, Suppl. 1.

3. Bosc, F. J.—*A Monograph on Cancer*, Paris, 1898.

of these bodies, which he considers to represent spore formation.

H. G. Plimmer[1] in his most recent article states having found these bodies in a large number of cancers, and gives illustrations from a cancer of the breast which contained unusual numbers. The figures on Plate I. (see Fig. 16), Figs. 7, 8, 9, 10 of Plate II. illustrate these inclusions.

Fig. 14.

Plate IV., Fig. 2, after Pianese.

Sjöbring[2] in his most recent publication, illustrates typical cell inclusions of especial interest. These are illustrated as apearing (see Fig. 17) in the epithelial cells of the epididymis of a rabbit, from the immediate neighborhood of a fragment of carcinoma which had been transplanted under sterile conditions into the tunica vaginalis of the animal.

E. von Leyden,[3] in Plate I., illustrates inclusions from a carcinomatous lymph node from carcinoma of mamma and a carcinoma of the cecum. He suggested the term "bird's-eye inclusion" which aptly describes the appearance of these structures.

Fig. 15.

Plate II., Fig. 4, after Bosc.

Gaylord[4] illustrates these bodies in Plates VIII., IX., XIV. and Figs. 2 and 3, Plate XV.; Figs. 1, 2, 3, and 4, Plate XVI., all taken from carcinoma of the breast.

Feinberg[5] describes characteristic bird's-eye inclusions embedded in the protoplasm of cancer cells, as well as a form

1. Plimmer, H. G.—On the Histology and Etiology of Cancer. The *Practitioner*, April, 899.

2. Sjöbring, N.—Ueber die Microörganismen in den Geschwülsten. *Centralblatt f. Bakteriologie*, Vol. XXVII., 1900.

3. Von Leyden, E.—Zur Aetiologie des Carcinoms. *Zeitschrift für klin. Medecin*, Berlin, 1901.

4. Gaylord, H. R.—The Protozoön of Cancer. *American Journal of the Medical Sciences*, May, 1901.

5. Feinberg.—Zur Lehre des Gewebes und der Ursache der Krebsgeschwülste. *Deutsche med. Wochenschrift*, XXVIII., 11, p. 185, 1902.

which he believes to be extra-cellular, which appears to be without a central body. His article is unaccompanied by illustrations

FIG. 16.

*a* Plate I., Fig. 5, after Plimmer.
*b* " I., " 6, " "
*c* " II., " 9-10, " "

but from examination of his specimens there is no doubt Feinberg in part deals with the typical inclusions.

Greenough[1] illustrates on Plate XXXIII., Figs. 2 and 3, what appear to be typical bird's-eye inclusions. Plate XXXV., Fig. 3, appears to contain a typical inclusion. Those shown on Plate XXXIII. were from a cyst adenoma of the breast. Fig. 3, Plate XXXV., is from carcinoma of the breast.

FIG. 17.

Fig. 1, after Sjöbring.

Nösske[2] illustrates on Figs. 1, 2, 3, 4*c* and 10, typical bird's eye inclusions.

E. von Leyden[3] illustrates on Plate I. (see Fig. 18) cells containing these inclusions from a case of pulmonary carcinoma and cancer of the breast. In Fig.

1. Greenough.—Cell Inclusions in Cancer. *Journal of Medical Research*, Vol. VII., No. 2.
2. Nösske.—Untersuchungen über die als Parasiten gedeuteten Zelleinschlüsse im Carcinom. *Deutsche Zeitschrift für Chirurgie*, Bd. LXIV.
3. Von Leyden, E.—*Ueber die Parasiten des Krebses*, 1902.

4*a* and *d* he shows a cell containing large masses of these inclusions, which he thinks are the termination of a process of spore formation. Figs. 3*a* and 3*b* show the same accumulation of closely packed smaller bodies in another cell. Several of his illustrations represent the bodies in the fresh state stained and unstained.

FIG. 18.

Plate I., Fig. 4*a*, after von Leyden.

Posner[1] has recently published five drawings made by himself in 1876 while a student of Wagner's. (See Fig. 19.) These represent characteristic bird's-eye inclusions from carcinoma of the breast and one illustration of characteristic bird's-eye inclusions in a sarcoma cell from sarcoma of the spinal cord. This is the first recorded observation of bird's-eye inclusions in sarcoma.

Klimenko,[2] in an article reviewing a previous article of Feinberg, describes bird's-eye inclusions which he thinks, however, are not identical with those described by Feinberg. He was unable to find an inclusion corresponding exactly to those given by Feinberg. He has attempted to determine by microchemic reactions whether the bird's-eye inclusions are the result of degenerative processes, and concludes that they give no microchemical results indicating that they are. He agrees with Nösske that the inclusions are probably the result of secretive and excretive activity, or perhaps even evidence of reserve food stuff (glycogen) in the cells. He observed the fact that in certain cases of carcinoma of the breast, where there was great metotic activity in the cells, but few inclusions were present. On the other hand, in certain carcinomata, in which proliferation was not markedly active, a large number of inclusions could be found. He was unable to determine a direct relation between

FIG .19.

*a* Fig. 3, after Posner.
*b* " 5, " "

1. Posner.—Notiz über vogelaugenänliche Einschlüsse in Geschwülstzellen. *Archiv. für klin. Chirurgie*, Bd. LXVIII., Heft 3.

2. Klimenko.—Eine Nachprüfung der Arbeit Dr. Feinberg's über seine Krebsparasiten Beitrag zur Frage über die Einschlüsse in und zwischen den Krebszellen. *Centralblatt für Pathologie*, Vol. XIII., No. 21, p. 837.

the presence of inclusions and metotic activity in the neighboring cells. He was never able to detect inclusions in cells undergoing division.

The theories advanced as to the significance of these bodies: (*a*) as degenerations of various kinds of the cytoplasm and nuclei of the cells. Of the authors already cited those who have undoubtedly observed and illustrated the inclusions which we are considering, Virchow, Pianese, Greenough and Nösske, interpreted them as the result of transformation or degenerations of the component part of the cells. Virchow in his first article interpreted these bodies as metamorphosed nuclei. In his second article he advanced the theory of endogenous cell formation and believed that the inclusions illustrated were cells thus formed. Borrel[1], who previously held that these bodies were of a parasitic nature, has recently suggested that they are produced by alterations in the centrosomes of the cancer cells. Pianese's article deals with a great variety of inclusions and only those quoted, Fig. 2, Plate IV., can be accepted as sufficiently characteristic to be included under the type we are considering. Pianese's investigations led him to the conclusion that all the structures illustrated were the result of different forms of metamorphosis, vacuolisation of the protoplasm, and hyaline and colloid degeneration. It would appear from the recent publication of Pianese that he has somewhat modified his interpretation of at least certain varieties of the inclusions described in his extensive monograph. In his most recent[2] article, which deals with a protozoon infecting the renal epithelium of the guinea pig, Pianese concludes that the presence of this organism leads to the development of active mitotic changes of the adjacent epithelial cells. These figures are either typical or atypical, and the latter closely resemble the forms of mitosis found in carcinoma. He likewise finds that the epithelium presents other characteristics (karyolysis, karyorrhexis, nucleorrhexis and nucleolysis) uniformly observed in cancer cells. Lastly, he finds that many of the epithelial cells contain inclusions closely resembling certain varieties found in carcinoma. He was unable to satisfy himself as to whether these inclusions were stages in the development of the parasite or changes produced in the cell by the indirect action of the organism. It

1. Borrel.—Les Theories parasitaires du cancer. *Ann. de l'Institut Pasteur*, Vol. XV., 1901.

2. Pianese.—Ueber ein Protozoon des Meerschweinchens. *Zeitschr. f. Hygiene und Infectionskrankheiten*, Vol. XXXVI., Part 3.

would appear from this article that Pianese does not at present hold fast to the conclusion, that all the cell inclusions in cancer are the result of degeneration of the cells.

Greenough concludes these bodies are the result of secretory activity of the cell. He bases his conclusions on the facts that the inclusions are not found in epithelioma or sarcoma, and that they are found in non-cancerous diseases of the mammary gland, *i. e.*, fibroadenoma. Nösske arrives at a conclusion similar to that of Greenough, believing the inclusions to be the result of secretory activity on the part of the cells. Borrel[1] interprets many of the cell inclusions as centrosomes.

Besides these authors a number of critical articles have appeared. Lubarsch[2] believes that the inclusions may be one of the four following: First, they may be the result of vacuolisation of the cell protoplasm in which protoplasmic remains become condensed and form a central body. Second, they may be centrosomes (Borrel), numbers of which have been found in giant cells by Heidenhain and Benda. Third, the bodies are the result of the phagocytic activity of the cancer cells which take up red blood corpuscles, these disintegrate, the remains forming the central body. Fourth, the bodies are caused by secretion granula around which a halo of pale protoplasm is formed. To these four may be added two more. Fifth, the belief that they are the result of special secretory activity on the part of the cells (Greenough, Nösske); and, sixth, Hansemann[3] believes that these bodies are formed as a result of the fixative which extracts the water from hyaline material embedded in the protoplasm. As a result of this extraction of water the hyaline material remains as a condensed central mass which forms the central body.

It will be seen from the above enumeration, that investigators who have interpreted these so characteristic inclusions as the result of metamorphosis of the cytoplasm, are in no way agreed as to the manner in which they are formed. Those who hold that the bodies are probably parasitic in their nature, have advanced certain arguments in objection to the above interpretation.

First of all, all investigators who have interpreted the bodies as parasites have failed to find characteristic inclusions in any

1. Loc. cit.
2. Lubarsch.—*Pathologische Anatomie und Krebsforschung*, 1902.
3. Hansemann.—Die mikroskopische Diagnose der bösartigen Geschwülste. Second Ed., 1902, Berlin.

other structures than tumors. It is claimed that in properly fixed sections the bodies present a characteristic appearance, and can be distinguished from simple vacuolisation of protoplasm. The most recent utterance on this subject is that of Benda.[1] He states that although he has searched carefully for these inclusions in all kinds of tissue he has failed to find them in anything but malignant growths. It must appear, then, that if the bodies are the result of vacuolisation of protoplasm, this form of degeneration is characteristic of carcinoma cells. An apparent exception to this statement is that contained in the publication of Nösske. This author claims to have found typical bird's-eye inclusions in the epithelium of the normal acini of the breast. An analysis of this statement shows first, that the normal breast referred to was located at the margin of a nodule of carcinoma. Through the courtesy of Prof. Marchand, the writer has had the privilege of seeing this section. After carefully viewing the preparation it appeared to us that the epithelium in question had already begun to proliferate. In one portion of the acinus, there were three distinct layers of the epithelium. We were of the opinion in this case that the epithelium had already undergone cancerous metamorphosis. Dr. Nösske exhibited this preparation at the 31st Surgical Congress in Berlin, April 3, 1902. It was viewed by a number of pathologists, a majority of whom were likewise of the opinion that the epithelium had already undergone carcinomatous transformation. The presence of the inclusions, therefore, at the margin of carcinoma nodules would rather more strongly indicate that they were of parasitic nature than that they were degenerations. The observation is not new, the distribution of the inclusions in the acini at the margin of carcinoma of the breast, having been referred to and accurately described by Cattle.

The bodies illustrated by Greenough found in fibroadenoma of the breast are undoubtedly characteristic bird's-eye inclusions, but inasmuch as fibroadenomata are known in many cases to become transformed into cancer, and as the etiology of this form of tissue development is unknown, their appearance in this class of tumor in no way excludes the possibility of their being parasites. The inclusions shown by Nösske, as appearing in the bronchial epithelium in a case of congenital syphilitic pneumonia in a new-born child, are so essentially different from the

1. Benda.—Sitzung der Karzinom Comite, Oct. 4, 1902.

bird's-eye inclusions as to be excluded, even from the illustrations which accompany his article.

As to the second possibility that the bodies are centrosomes, as noted by Benda and Heidenhain (Borrel and LeCount[1]), this supposition is rendered improbable by the great number occasionally found in a single cell. Kürsteiner counted as many as 80 in one cell, and Plimmer[2] illustrates a cell containing 27 inclusions. (See plate Fig. 2.) Accompanying this article illustrates a cell in which 18 cell inclusions can be counted. Secondly, the fact that centrosomes can be found in cells containing these inclusions and can be definitely distinguished from them should dispose of this interpretation of the body.

In this connection, the evidence of Benda (*Verhandlungen der deutschen Gesellschaft für Chirurgie*, 31st Surgical Congress, Berlin, April 3, 1903, p. 73,) is of great importance. One of the first to describe centrosomes, and quoted by Lubarsch as an authority, he states first that centrosomes have often been sought for in carcinoma cells without their presenting any pathological alterations. He states that he has recently, with new and specially proved methods, investigated this question, and holds that the *theory of Borrel is incorrect*, in that he has repeatedly found the centrosomes intact in cells which contained numbers of the bird's-eye inclusions. He holds that the hope of explaining the abnormal proliferation of carcinoma cells as the result of alterations of the centrosome is fruitless.

Third, the interpretation of these bodies as disintegrating red blood cells taken up by the epithelium (Lubarsch) has been advanced by various observers. This criticism has likewise been applied to preparations of our own in which we had injected plasmodiophora spores into the tissue of warm-blooded animals, in repeating the experiments of Podwyssozki[3] where plasmodiophora spores were taken up by the phagocytic connective tissue cells of the animal and presented an appearance, in many cases, indistinguishable from the bird's-eye inclusions in carcinoma. In attempting to definitely exclude the red blood cells as a factor in the production of these forms, which we held to be metamorphosed spores, we examined a number of specimens taken from nodules developing on the peritoneal surface of frogs which had been inoculated in the same way. These

1. *Journal of Med. Research.*

2. Loc. cit.

3. Podwyssozki.—Myxomyceten, resp. Plasmodiophora Brassicæ Woron. als Erzeuger der Geschwülste bei Tieren. *Centralblatt für Bakteriologie*, Vol. XXVII., p. 97.

nodules were composed of endothelial cells (as described by Podwyssozki) containing the spores of plasmodiophora brassicæ, many of which again presented an appearance identical with the cell inclusions in carcinoma, and identical with the spores embedded in the connective tissue phagocytes surrounding the fragments of implanted clubroot in warm-blooded animals. (See Plate, Fig. 2.)

The results of these two lines of experimentation were exactly the same, but the great difference in the red blood corpuscles of the warm-blooded and cold-blooded animals, in the first case non-nucleated, in the second case much larger and nucleated, left no doubt that the inclusions in both cases were the metamorphosed spores of plasmodiophora brassicæ. In neither series of experiments were we able to determine that the phagocytes had taken up red blood cells, although in other pathological conditions, *i.e.*, typhoid fever, it is a recognised fact that phagocytic cells commonly take up the erythrocytes. The difficulty of determining the origin of inclusions in cells without the aid of experimentation is emphasised in this case. Many of these bodies are larger than red blood corpuscles, and in properly prepared specimens we have never been able to find any ground for holding that they were altered erythrocytes. The fact that in many cases the central bodies of the bird's-eye inclusions can be stained with a nuclear stain, likewise speaks against this supposition.

Fourth and fifth, that the bodies are not secretion granula or the result of special secretory activity on the part of the cell is more difficult to prove, but the fact that they have occasionally been found (Posner) in sarcomata renders either explanation rather improbable. The statement of Nösske that they are only found in glandular epithelium and almost exclusively in the mammary gland, a view which Greenough holds, and to which we were previously inclined ourselves, is shown to be incorrect by a critical review of the literature of the earlier writers. This shows that undoubted bird's-eye inclusions have been found in carcinoma arising from other types of epithelium.

Sixth, that the characteristic appearance of these bodies is not due to any action of the hardening process (Hansemann) is shown by their *constant appearance in fresh material, where they have later been found in hardened and stained sections.*

As parasites.—With the exception of the authors above mentioned, the remainder of those who have accurately described or illustrated these inclusions have interpreted them as parasites.

The earlier investigators compared them to recognised types of protozoa, notably the coccidia and the gregarinæ. Many of these authors describe, besides the forms which we are considering, larger and more complex structures presenting appearances not unlike certain forms in the development of these organisms. The younger forms of coccidium perforans (Leuch) (coccidium oviforme), in the epithelium of the intestinal tract of rabbits is extremely like in appearance the bird's-eye inclusions of cancer.[1] The readiness with which ectogenous forms of coccidia can be detected in the tissues of the animals infected, has, however, weakened the force of this comparison, because of the absence in the fresh material of cancer of forms presenting anything like the definite characteristics of the ectogenous cycle of the coccidia. A class of organisms which has attracted the attention of the more recent writers on the subject is the mycetozoa, especially one of the group, plasmodiophora brassicæ, an organism which causes tumors and outgrowths in plants. This class was previously placed among the lower types of fungi, but more recently has been accepted as a variety of protozoa. Doflein, in his recent book on the subject classifying it under the rhizopoda.

The first to call attention to a possible connection between cancer and kohlhernia was R. Behla. He noted the prevalence of this disease in plants in localities in which cancer was prevalent. He inoculated animals with fragments of the plant tumors and noted that granulation tumors resulted. He did not, however, detect the presence of the parasite in the tissue cells. Podwyssozki[2] after viewing the preparations of Nawaschin in a preliminary announcement, called attention to the fact that certain forms of this organism and certain inclusions in cancer presented great points of similarity. He afterward reported experiments upon rabbits, guineapigs, frogs and axolotls, in which he had succeeded in producing infection and had detected the presence of the spores in the cells of the infected animal. He found that the introduction of the spores of plasmodiophora in the tissues of both cold and warm-blooded animals, led to the development of tumors of considerable size made up of cells derived, either from the connective tissue, or from the endothelium of the surrounding lymph spaces. In the latter case the tumors presented the characteristics of an endothelioma. He

1. See Fig. 221, Lang, Lehrbuch der vergleichende Anatomie der wirbellosen Thiere. Zweite Lieferung. Protozoa.

2. Loc. cit.

was able to determine that the presence of the parasite led to the proliferation of the nuclei of the infected cells.

Following Podwyssozki, von Leyden[1] likewise noted the similarity between bird's-eye inclusions and the ameboid form of plasmodiophora in the plant cell. Feinberg[2] emphasises the same similarity. Nösske does not think the similarity of the cell inclusions and the ameboid form of plasmodiophora to be so great.

All these comparisons, except that of Behla are based upon the publication of S. Nawaschin.[3] Upon receipt of this article we were greatly surprised at the remarkable similarity between many of Nawaschin's figures and bird's-eye inclusions in cancer. According to Nawaschin, the organism first appears in the plant in the form of an ameba, after having entered as a swarmer. These amebæ are found in the plant cells lying in the sap spaces and, except in very early infections, a number were found in each cell. The amebæ contain in their protoplasm when observed in the fresh state, highly refractive granules of equal size, which present an appearance very much like fat.

FIG. 20.

Plate XX., Fig. 6, after Nawaschin.

The nuclei are spherical or oval and consist of a vacuolous structure with a central body. It is this nucleus which so resembles the bird's-eye inclusions. The organism having become transformed into an ameba in the plant cell, *lives a strictly symbiotic existence with the infected cell.* It divides by a special form of cell division which we shall consider in detail, and only when the plant cell is exhausted is the cycle inaugurated, which leads to spore formation.

This latter cycle, that of spore formation, is entirely distinct from the process of nuclear division by which the amebæ increase in number. In the younger forms of the organism (Fig. 20), the protoplasm surrounding the nucleus of the parasite is scarcely distinguishable from the protoplasm of the plant cell and extends into it in delicate prolongations. The change

1. von Leyden, E.—Zur Aetiologis des Carcinoms. *Zeitschr. f. klin. Medecin*, Vol. XLIII., Part ½. Ueber die Parasiten des Krebses. *II. Ergänzungsband vom klinischen Jahrbuch.*

2. Feinberg.—Ueber den Erreger der krankhaften Auswüchse des Kohls (Plasmodiophora Brassicæ Woronin). *Deutsche med. Wochenschr.*, March 3, 1902.

3. Nawaschin, S.—Beobachtungen über den feineren Bau und Umwandlungen von Plasmodiophora Brassicæ Woron. im Laufe ihres intracellularen Lebens. *Flora*, 1899, Vol. LXXXVI., Part 5.

leading to nuclear division in the ameba, are first found in the appearance of fine grains of chromatin within the nucleus, scattered indiscriminately in the clear space between the central body and the periphery. (Fig. 21*a*.) The nuclei in this stage are somewhat larger than immediately after division. These chromatin granules group themselves about the central body which first becomes indistinct (Fig. 21*b*), and then divides. They are more or less definitely arranged in the form of a circle in the plane of the division. (Fig. 21*c*.) A section through the nucleus in this stage shows the two central bodies closely approximated, with the smaller chromatin granules in the transverse axis. A section in the transverse axis shows the central body surrounded by a ring of chromatin granules, presenting the appearance of a rosette. (Fig. 21*d*.) The nucleus

FIG. 21.

*a* Plate XX., Fig. 14, after Nawaschin.
*b* " XX., " 17*f*, " "
*c* " XX., " 17*e*, " "
*d* Plate XX., Fig. 17*d*, after Nawaschin.
*e* " XX., " 18*d*, " "
*f* " XX., " 15, " "

in this stage has already assumed an oval form and the central bodies then withdraw to the poles of the cell. (Fig. 21*e*.) In this stage they are sometimes connected by one or two threads of chromatin which later disappear. Division is then completed by the capsule folding in at the center, leaving two perfectly formed nuclei each with a central body, the two closely approximated. (Fig. 21*f*.) In this way a single ameba may possess a number of nuclei.

Aside from the characteristic nucleus, the protoplasm of the ameba is of interest, containing, as it does, in the fresh state, coarse granules of uniform size. These granules are blackened by osmic acid and on the extraction of the fat from the preparations they disappear, leaving a coarsely reticulated protoplasm. (Fig. 21*f*.) The central body of the nucleus, when stained with Flemming's triple stain, reacts variably to the stain. When in the ameboid form the central body of the nucleus stains brilliant red. In the first stage of spore formation, the central body stains a deep blue-black. This point is of importance inasmuch as certain discrepancies in the staining of the central bodies of the bird's-eye inclusions have been previously noted.

The process which leads to spore formation is entirely distinct from that by which the ameba, as such divides. In preparation for this process the nuclei of the ameba divides repeatedly by the method already described, thus each ameba contains a number of small nuclei. The margins of the ameba then become indistinct, the nuclei likewise become less distinct and the different amebæ coalesce. The central body of the nucleus then becomes very indistinct, the protoplasm granular. The nuclei at this stage contain fine granules and are with difficulty distinguished from the surrounding protoplasm. The whole structure now forms a plasmodial mass. There now develops in each nucleus a well-defined spindle with medullary plate, all of the nuclei dividing at once. This process of division follows the recognised types of mitosis and when complete the entire mass breaks down into small amebæ, each containing one nucleus. These are at first of irregular shape and stain poorly. The irregularly shaped myxamebæ are gradually rounded and condensed until they ultimately form spherical bodies of similar size with well-defined margins, each containing a highly refractive central body. It is to be noted that *the complete spore again presents an appearance not unlike the nuclei of the amebæ.*

Recognising the great similarity between the bird's-eye inclusion in cancer cells and the nuclei of the ameba in plasmodiophora in the plant cell, a similarity which to a lesser degree is shared by spores of plasmodiophora, it occurred to us to search our preparations for evidence of some form of division in the inclusions, and likewise to determine if the protoplasm surrounding the inclusions could be distinguished from the remaining protoplasm of the epithelial cell. In searching our preparations we came upon one which has already served us for photography, the illustrations of which appear in our previous publication, being the low power illustrations on Plate VIII., Plate XIV., Fig. 1, Plate XVI.[1]

This preparation consisted of a thin section, approximately 2 mm. square, taken from the margin of a rapidly growing carcinoma of the breast. It contained a large number of inclusions, in many fields every cell containing one or more. The fixation of this preparation was unusually successful and the differentia-

1. *American Journal Med. Sciences.*

tion excellent. We had little difficulty in repeatedly finding forms extremely suggestive of the method of division, illustrated in Fig. 21—*a*, *c*, *d*, *e* and *f* from Nawaschin. Fig. 3, Plate I., was repeatedly found and appears to represent a stage similar to that shown in Fig. 21*a* Nawaschin. We next repeatedly encountered inclusions in which the central body had divided and in nearly every case found two small granules of chromatic material in the transverse axis so arranged as to present an appearance exactly like Fig. 21*e* from Nawaschin. In Fig. 4, of Plate I., an inclusion presenting this appearance is shown. The two granules in the transverse axis are extremely small and were photographed with difficulty. It is this form which, when sectioned in the transverse axis, produces the rosette appearance which was likewise repeatedly encountered. In Fig. 5 of Plate I., the central body lies somewhat below the plane of focus and the rosette appearance is not so characteristic as occasionally seen. Many forms were found in which the rosette was apparently sectioned in an oblique plane so that but one half of the rosette appeared in the inclusion. Fig. 6, of Plate I., shows what appears to be the next stage in the process of division, the two central bodies having withdrawn to the poles of the cell, and separation of the two by the capsule is suggested by the appearance of the inclusion. Bodies which had apparently divided were repeatedly found, Fig. 7, of Plate I., representing this stage. In Fig. 8, of Plate I., are likewise seen bodies cut somewhat obliquely, also representing this stage. We had no difficulty in finding all of these forms repeatedly.

A careful examination of the protoplasm surrounding the inclusions was made to determine whether a differentiation of the possible protoplasm of the parasite and that of the epithelial cell could be made. In many cases a distinct reticulation of the protoplasm was visible as in that surrounding the two inclusions in Fig. 7, of Plate I. In one or two portions of the section we found what appeared to be inclusions surrounded by reticulated protoplasm which had become detached from the nuclei of the cells in which they were included. Fig. 8, of Plate I., shows an inclusion of this sort surrounded by reticulated protoplasm, which has become detached from the nucleus of the cell lying slightly to the left. In certain cells we found large numbers of small inclusions closely packed. In these we were unable to find any evidence of the changes above described. Fig. 2, of Plate I., shows a cell containing a number of bodies somewhat more distinct than those containing the apparent nuclear changes.

A number of observers have undoubtedly spoken of similar groups of inclusions, as representing spore formation. (Bose, von Leyden, Jackson, Clarke.) The similarity of inclusions of the type shown in Fig. 2, of Plate I., to those already described, led us to repeat the experiments of Podwyssozki, for the purpose of ascertaining what appearance the spores of plasmodiophora brassicæ presented when embedded in the connective tissue and phagocytic cells of animals. This series of experiments was undertaken with uniform results. The spores could be found in the large connective tissue phagocytes embedded in the protoplasm of the cells. They consisted for the greater part of spherical bodies of vacuole-like appearance with a central body. Hardened and stained with Plimmer's method, they stained, as does the protoplasm, and presented an appearance in size, form and other characteristics indistinguishable from the cell inclusions in cancer. Fig. 1, of Plate I., represents two large phagocytic cells from the margin of an implanted fragment of clubroot in a guinea-pig, removed after nineteen days. The cell to the left contains a form of inclusion most commonly seen. Between the two cells are two larger forms which are constantly encountered in the fresh scrapings. These are spherical bodies with delicately defined limiting membranes and with fine granular contents or in some cases filled with homogeneous material. The two bodies shown lying directly between phagocytes are undoubtedly of this nature. It is probable that they represent a stage in degeneration of the spores. They are of interest inasmuch as bird's-eye inclusions in cancer are commonly encountered, which contain finely granular material and have invariably been quoted in support of the degeneration theory. It is evident that these spores under certain conditions undergo degeneration, which would render their differentiation from other products of degeneration difficult. Nösske has recently advanced a line of reasoning of this nature to show that all the bird's-eye inclusions represent a form of secretory activity on the part of the cells. Those who have studied the bird's-eye inclusions have apparently failed to consider the possibility of the inclusions, if of parasitic origin, undergoing a process of degeneration.

It will be seen from the foregoing that in the spores of plasmodiophora brassicæ, we have a form of parasite which, embedded in the protoplasm of a cell, frequently presents an appearance indistinguishable from certain of the cell inclusions in cancer. It must, therefore, be conceded that the inclusions in cancer which so *closely resemble them are possibly of parasitic origin. Furthermore, the detection in the inclusions in a single slide, of a cycle of changes so closely representing the process of nuclear division in the ameboid form of plasmodiophora, must be viewed as strongly indicating the parasitic nature of these inclusions.*

Some of the phases of what we view as a process of nuclear division in the bird's-eye inclusions are indicated in the illustrations accompanying the articles of Soudakewitsch, Borrel and Plimmer. Fig. 2, *b*, which is Fig. 2, Plate VI., from Soudakewitsch, shows a body to the left and above which corresponds with Fig. 3 of the text from Nawaschin. Fig. 2, *c*, which is Fig. 7, Plate XII., from Soudakewitsch, represents a group of bodies nearly all of which present typical rosette forms. Fig. 2, *a*, which is Fig. 17, Plate XII. from Soudakewitsch shows a body to the left with two central bodies apparently in the act of dividing. Fig. 8, from Borrel, shows a large epithelial cell containing a number of inclusions, some with single central bodies, but a majority showing typical rosette forms. Fig. 16, *a*, from Plimmer (loc. cit.) shows an inclusion with central body surrounded by a number of grains of chromatin. Fig. 16, *b*, from Plimmer, shows a typical rosette form and Fig. 16, *c*, from Plimmer, shows three inclusions, two of typical rosette form and one with four bodies arranged in a manner not unlike Fig. 4, from Nawaschin. Under the head of Methods of Division, Plimmer (loc. cit.) states that the division into two is by far the most usual method of multiplication. This is effected, he believes, by a form of budding, or the nucleus becomes somewhat oval in shape and then divides into two parts. These soon become equal and separate, and then the capsule throws in septa from either side between the divided nucleus, which meet. They then separate from each other and become two separate individuals. The rosette form Plimmer interpreted as evidence of a form of division in which portions of the nucleus have become detached and arranged at the circumference of the organism. This he believes was followed by a process of segmentation with the formation of a number of parasites. With reference to the statement made by Plimmer that these bodies divide by a form of budding, we have never been able to detect any appearance confirming this observation. We are of the opinion that the rosette form corresponds closely to that described by Nawaschin and shown in Fig. 5 of the text.

CONCLUSIONS.—In summing up we would conclude that the spores of plasmodiophora brassicæ in the phagocytic cells of both warm and cold-blooded animals, when fixed and stained with the methods employed in the investigation of bird's-eye inclusions in cancer, under certain conditions, present an appearance indistinguishable from these inclusions. Second, that a series of changes can be found in the bird's-eye inclusions in cancer closely resembling the process of nuclear division, as described by Nawaschin in the ameboid form of plasmodiophora brassicæ.[1]

### DESCRIPTION OF PLATE.

FIG. 1. Large phagocytic cells from guinea pig, found at margin of implanted clubroot nineteen days after implantation. At *a* plasmodiophora spore embedded in protoplasm of cell. Central body of spore eccentrically placed. No condensation of cell protoplasm surrounding spore. At *b* two larger forms of spores containing protoplasmic masses (possibly partly developed swarmers).

Figs. 2 to 8 are taken from one slide, carcinoma of the breast, hardened and stained with Plimmer's method. Magnification of all figures, 1 to 8, × 940.

FIG. 2. Epithelial cell from carcinoma of the breast, containing 14 round cell inclusions of like size and appearance. Same magnification as Fig. 1 (Compare with *a*.)

FIG. 3. Cancer cells, containing large inclusions with well developed central bodies. Scattered between central body and periphery of inclusions numbers of chromatin granules. (Compare with first stage of division of intracellular ameba of plasmodiophora as shown by Nawaschin.)

FIG. 4. Elongated cell inclusion with two central bodies closely approximated. In the transverse axis two smaller grains of chromatin. (Compare with Fig. 2 from Nawaschin.)

FIG. 5. Cross section of inclusion similar to that shown in Fig. 4. The central body lies just below the focal plane, the grains of chromatin arranged in the form of a rosette about it. (Compare with Fig. 3 from Nawaschin.)

FIG. 6. Oval inclusion with two central bodies widely separated at poles. Suggestion of division with two bodies barely visible. (Compare Fig. 4, Nawaschin.)

FIG. 7. Two inclusions immediately after division. Central bodies well defined. Inclusions surrounded by reticulated protoplasm.

FIG. 8. Group of cells containing inclusions. At *a* an inclusion surrounded by reticulated protoplasm has become detached from the cell in which it was embedded. The inclusion in the surrounding reticulated protoplasm presents an appearance extremely like the myxamebæ of plasmodiophora.

---

1. On the point of sending this article to press, we have received a short epitome in the *Deutsche Medizinal-Zeitung*, Beilag für Karzinomlitteratur, of an article by S. Prowazek, entitled "Zur Kernteilung der Plasmodiophora Brassicæ Woronin." It is impossible to judge from the epitome to exactly what conclusions the author has arrived, but it would appear that he has questioned the significance of the changes described by Nawaschin.

## Figures and Tables

**Fig. I. f1:**
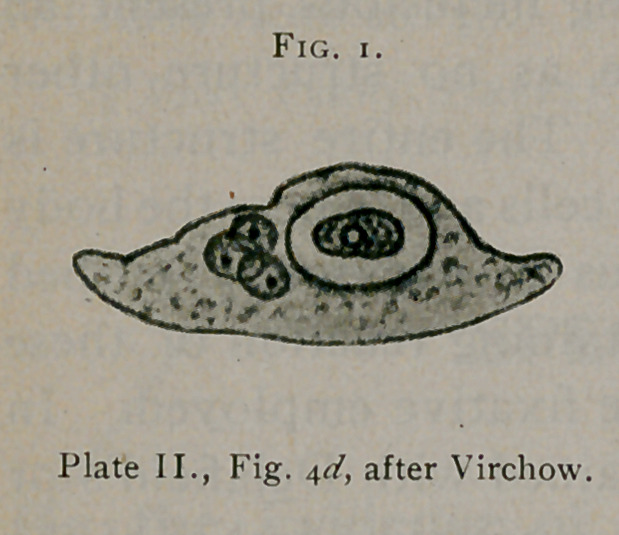


**Fig. 2. f2:**
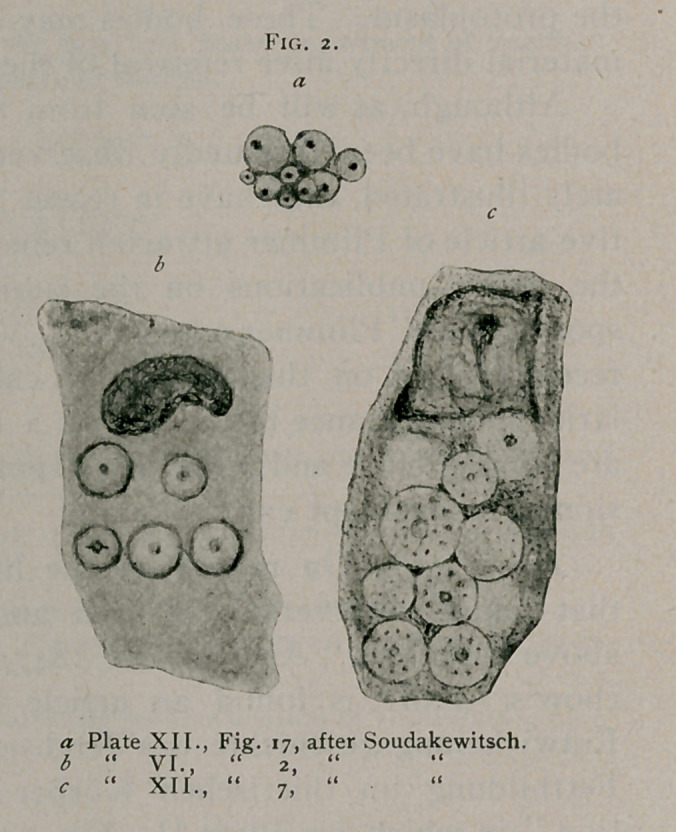


**Fig. 3. f3:**
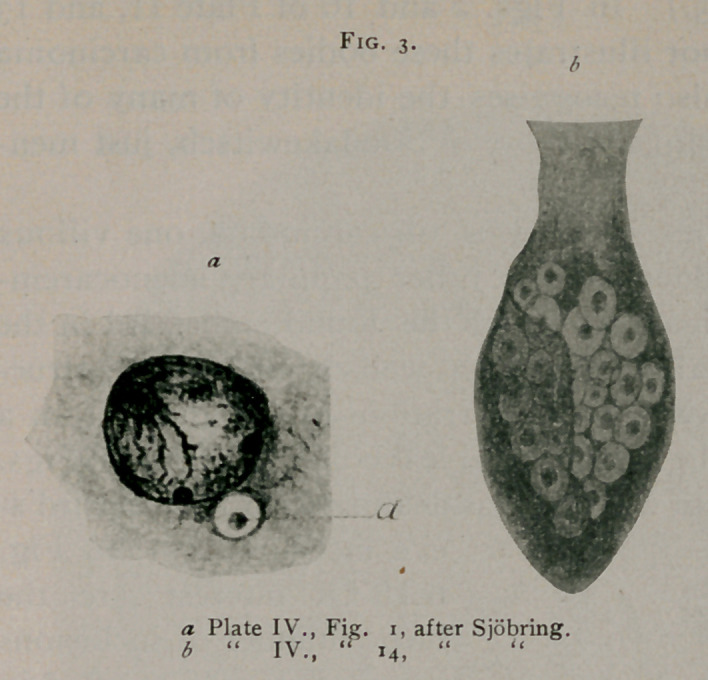


**Fig. 4. f4:**
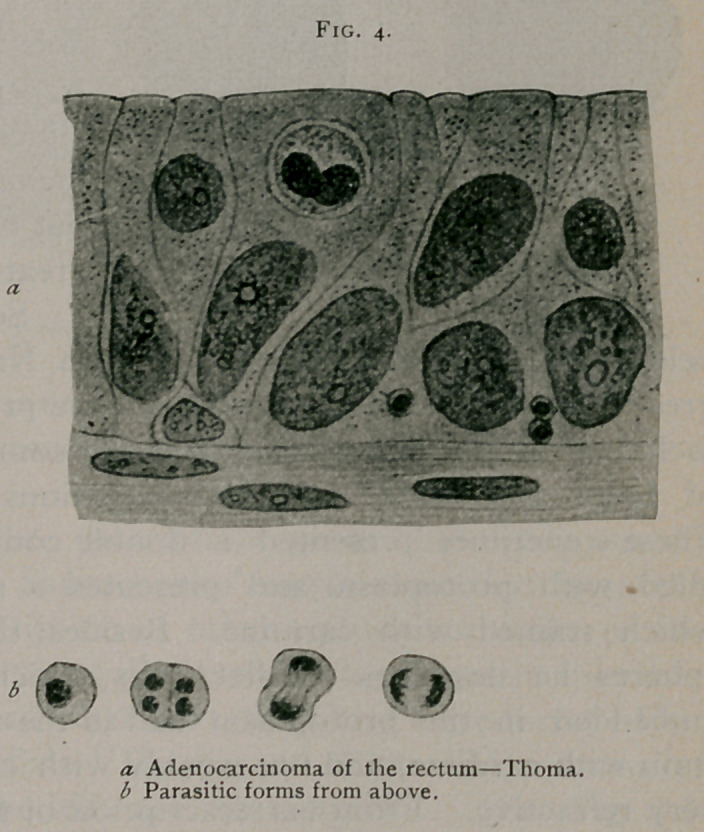


**Fig. 5. f5:**
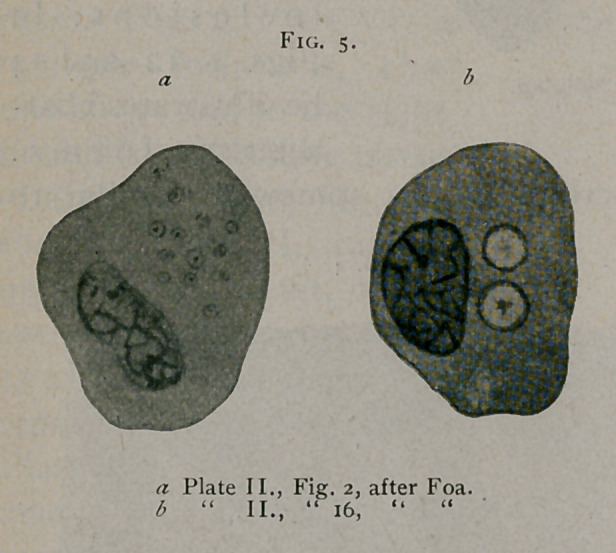


**Fig. 6. f6:**
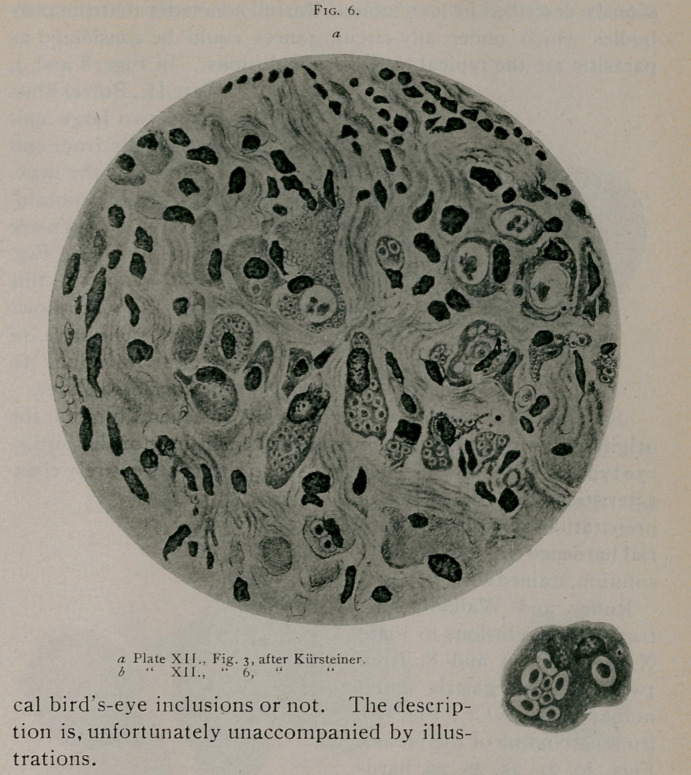


**Fig. 7. f7:**
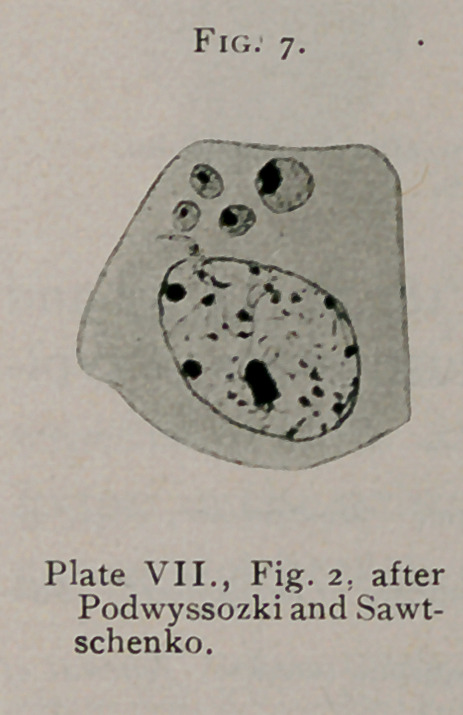


**Fig. 8. f8:**
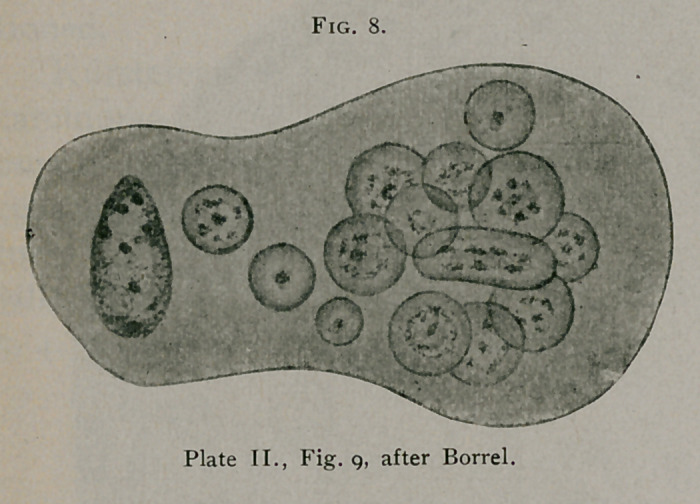


**Fig. 9. f9:**
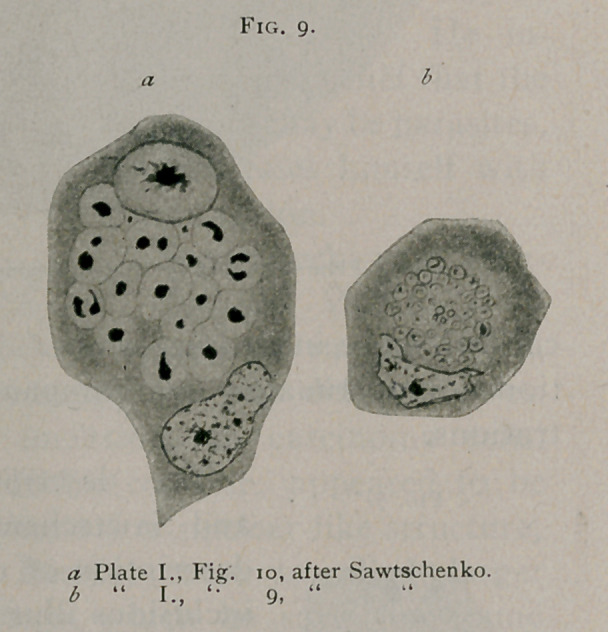


**Fig. 10. f10:**
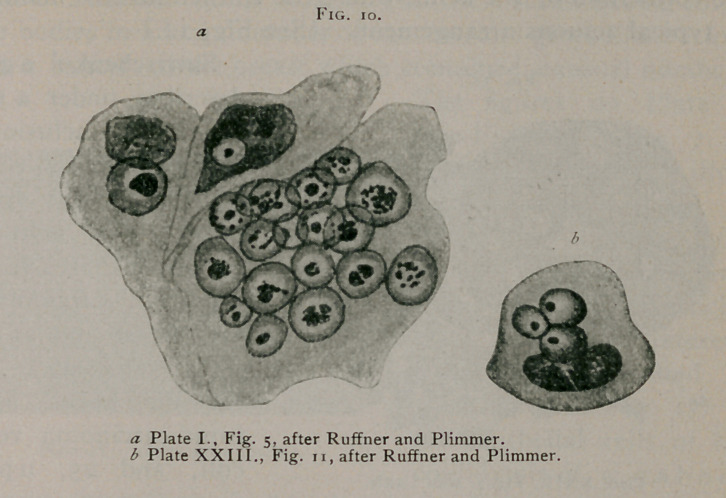


**Fig. 11. f11:**
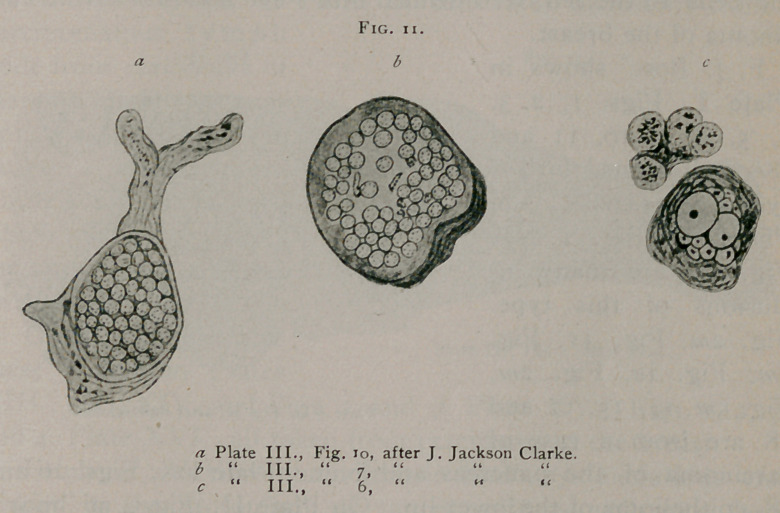


**Fig. 12. f12:**
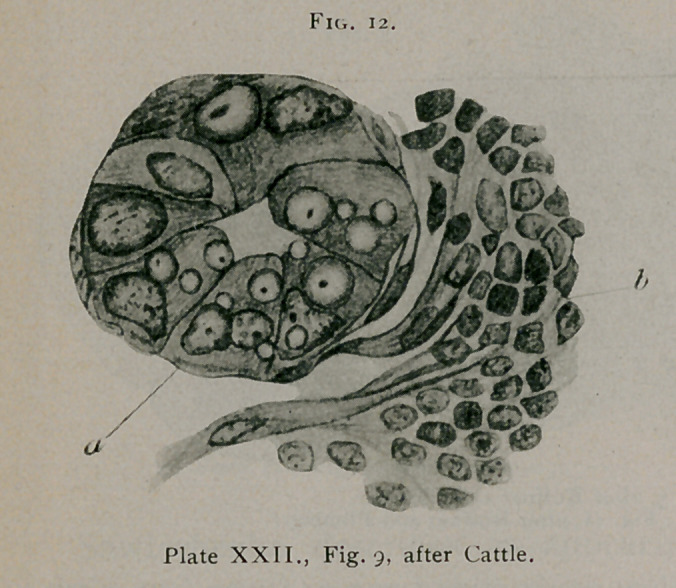


**Fig. 13. f13:**
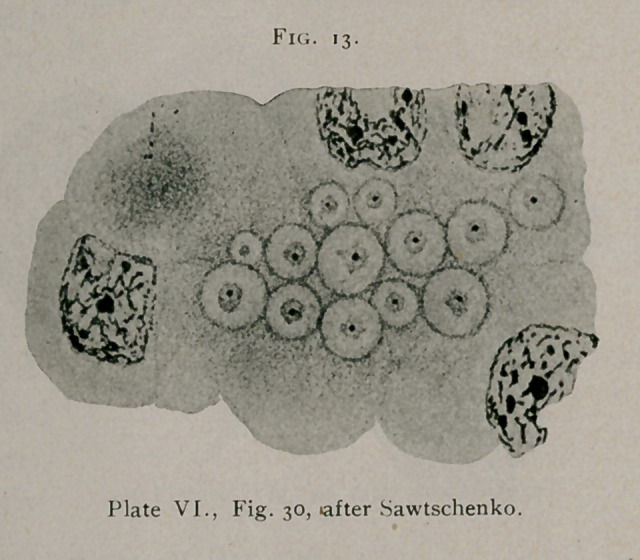


**Fig. 14. f14:**
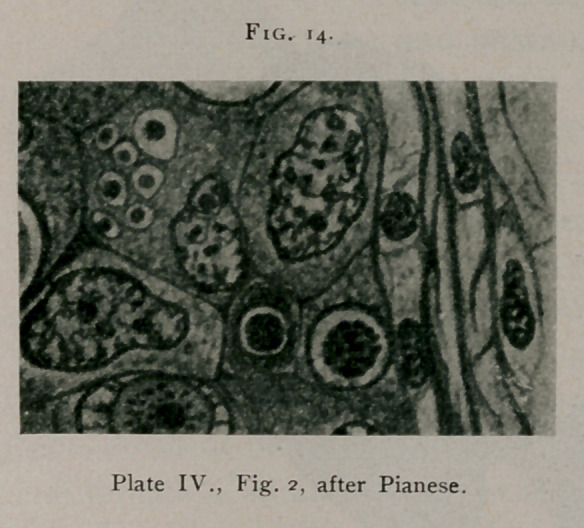


**Fig. 15. f15:**
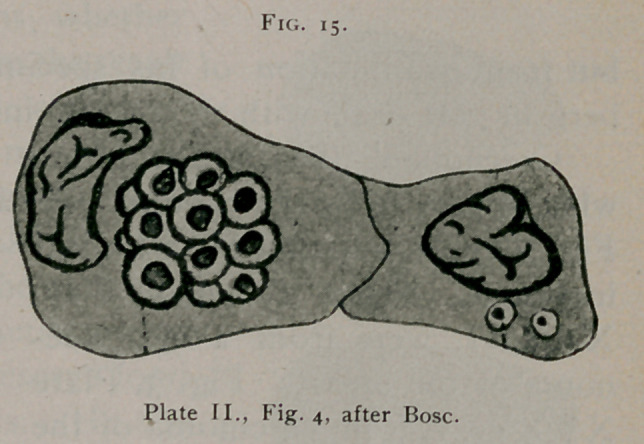


**Fig. 16. f16:**
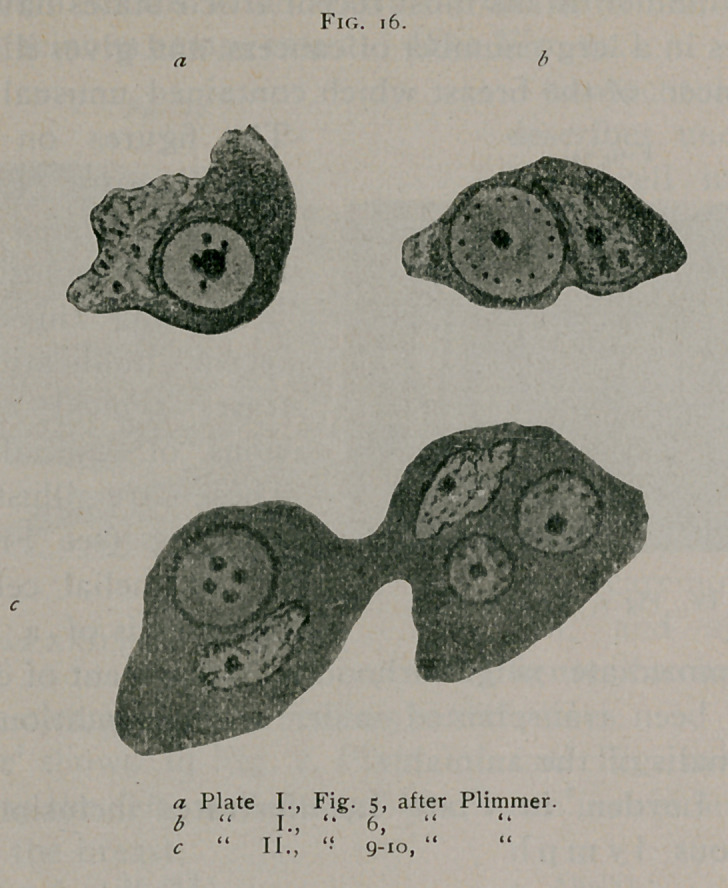


**Fig. 17. f17:**
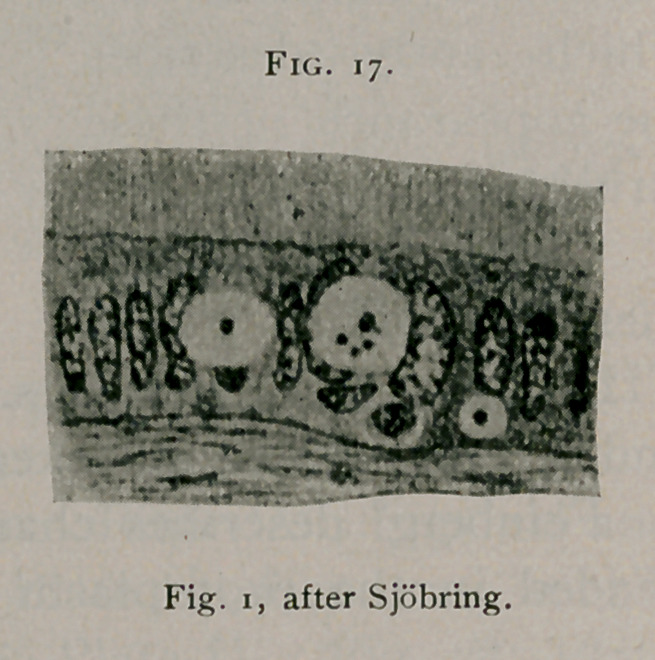


**Fig. 18. f18:**
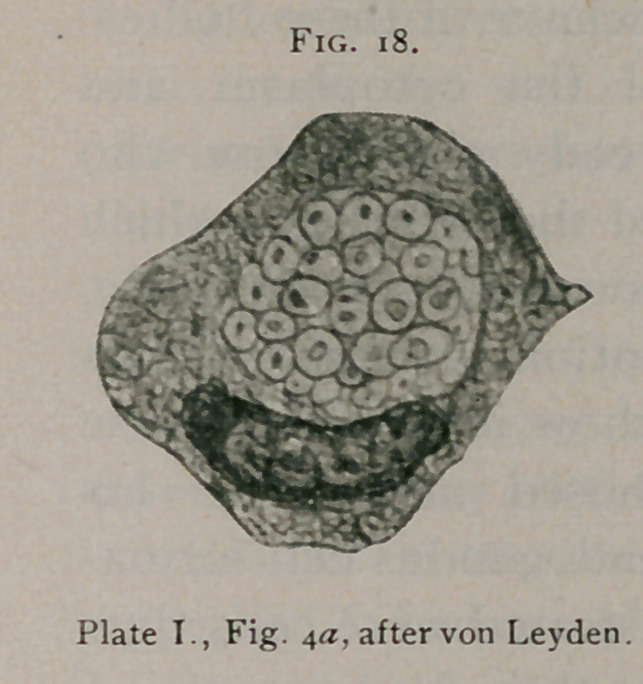


**Fig. 19. f19:**
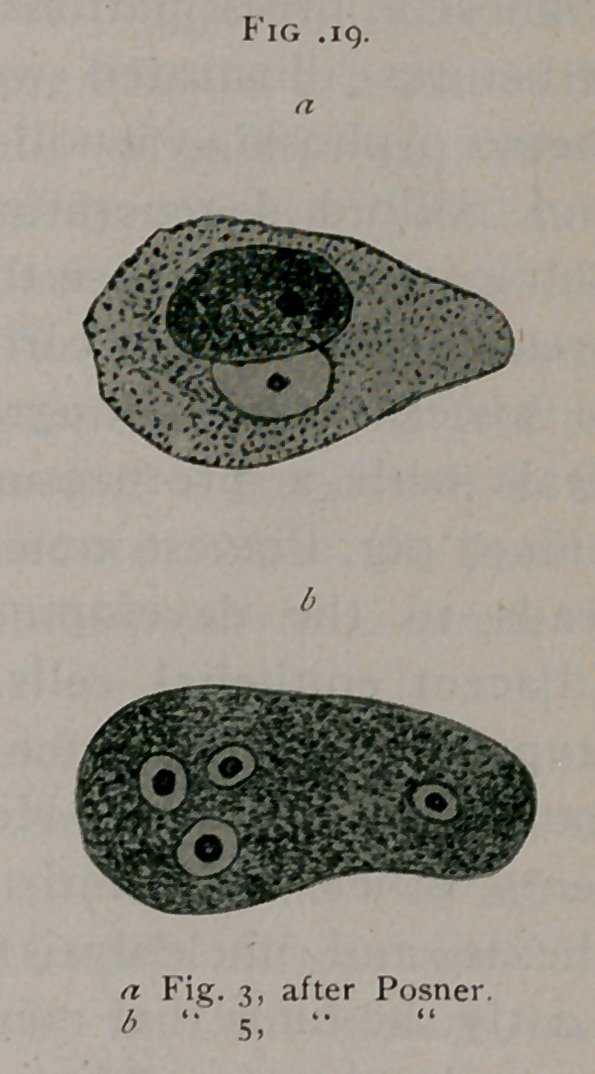


**Fig. 20. f20:**
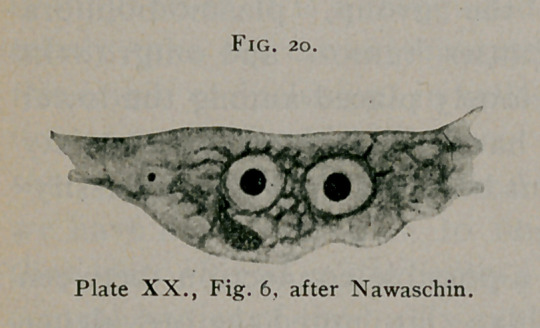


**Fig. 21. f21:**
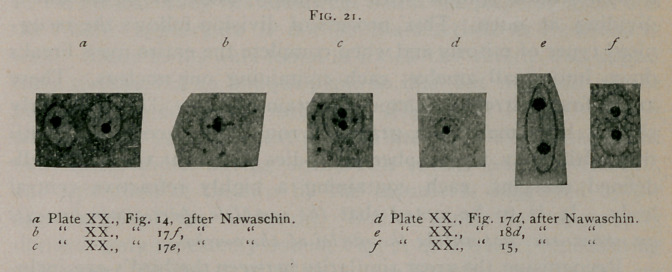


**Plate f22:**